# Irreducible Hernia With Unusual Content

**DOI:** 10.7759/cureus.31162

**Published:** 2022-11-06

**Authors:** Tharun G Chitrambalam, Nidhi George, Pradeep J Christopher, Koshy M Panicker, Sidhu Sekhar

**Affiliations:** 1 General Surgery, SRM Medical College College Hospital and Research Centre, Chennai, IND; 2 General Surgery, SRM Medical College College Hospital and Research CentreTRE, Chennai, IND

**Keywords:** diverticula, inguinal, hernia, bladder hernia, bladder diverticulum

## Abstract

Chronic increase in the intravesical pressure secondary to bladder outlet obstruction can lead to the formation of bladder diverticulum. Bladder diverticulum may get pulled into the hernial sac and may become a component of the hernia. Here, we report the case of an elderly male who had an unusual presentation of urinary bladder diverticulum as the content in an obstructed inguinal hernia. Upon exploration, the bladder diverticulum was released from the inguinal canal and returned to the peritoneal cavity, following which conventional hernioplasty was done. Inguinal herniation of bladder diverticulum is an uncommon condition and can be perilous during surgery if not diagnosed preoperatively.

## Introduction

Groin hernia is a common surgical condition and its content is usually intra-abdominal viscera surrounded by the peritoneum. An extra peritoneal organ cannot be contained in the sac of the hernia. However, it can be pulled by the sac itself and becomes a component of the hernia. Herniation of the urinary bladder is a relatively uncommon but not a rare condition. The diagnosis of urinary bladder as content is suspected if the size of hernia is variable on urination. The bladder can partially or entirely herniate into the inguinal canal and may cause obstruction, calculi, vesicoureteral reflux, hydronephrosis, infection, and acute renal failure. Long-standing increase of the intravesical pressure resulting from urinary bladder outlet obstruction can cause both secondary bladder diverticula and groin hernias. In rare cases, a diverticulum can be pulled by a hernia sac and becomes a component of the hernia itself. Such cases were encountered in inguinal, perineal, and obturator hernias. Here we report an unusual presentation of urinary bladder diverticulum as content in an obstructed inguinal hernia.

## Case presentation

**Figure 1:**
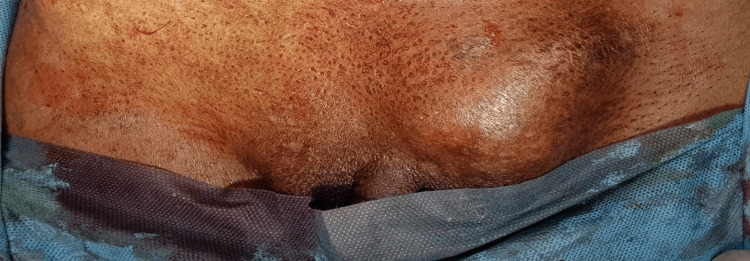
Tender, irreducible swelling in the left inguinal region

**Figure 2:**
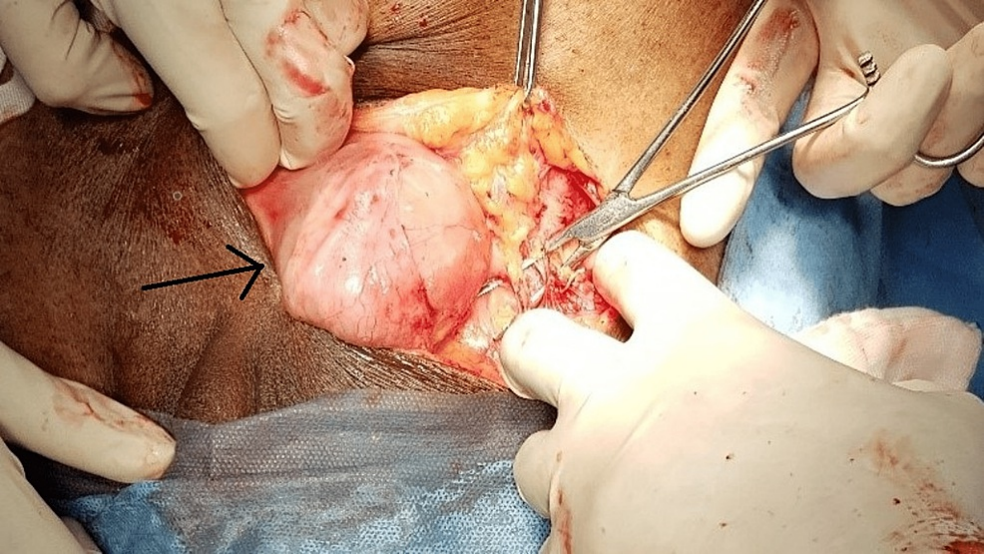
The hernial sac being separated from the cord structures and opened to discover urinary bladder diverticulum as the obstructed content

**Figure 3:**
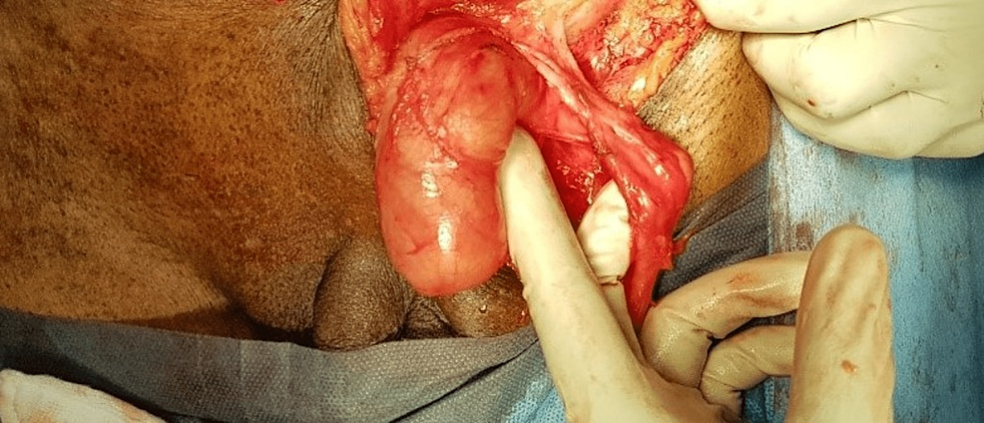
The bladder diverticulum dissected and freed from the inguinal canal

A 76-year-old male presented to the surgery outpatient department with complaints of a swelling in the left inguinal region for the past six months, associated with pain for the past two days. He also complained of nausea, vomiting, and constipation but was free of any urinary symptoms. The patient had undergone right inguinal hernia repair 30 years previously. The patient did not have any comorbidities and reported no other past medical history, no smoking history, or relevant family history. On examination, the abdomen was soft with a tender, irreducible swelling in the left inguinal region of size 3 x 3 cm (Figure 1) without an expansile cough impulse. Preoperative blood reports as well as urine analysis were well within the normal limits. The serum prostate specific antigen value was reported as 3.5 ng/ml. Ultrasonogram of the abdomen revealed a 3 x 3 cm defect in the external inguinal ring with herniation of urinary bladder. The prostate gland was mildly enlarged in size (4.1 x 3.2 x 4.4 cm) with a volume of 31 cc. Due to the presence of free fluid surrounding the hernial sac and the absence of cough impulse, possibility of obstruction/strangulation was considered and the patient was taken up for emergency inguinal exploration. The patient was catheterized preoperatively. Under spinal anesthesia, an inguinal skin crease incision was made, following which the external oblique aponeurosis was dissected to reveal the hernial sac. The sac was separated from the cord structures and opened to discover urinary bladder diverticulum as the obstructed content as shown in Figure 2. The bladder diverticulum was dissected, freed from the inguinal canal, and found to be inflamed but viable (Figure 3). The incision was extended and the urinary bladder was thoroughly inspected. Following this, the released bladder diverticulum was returned to the peritoneal cavity and the excess hernia sac was excised and reduced. The transversalis fascia was reapproximated and posterior wall plication was done. A polyproylene mesh of size 12 x 7 cm was placed and hernioplasty was completed. The procedure was well tolerated by the patient. He was discharged on the fourth postoperative day following Foley’s catheter removal, which was done on the second postoperative day. Following catheter removal, the patient did not report any urinary symptoms and was voiding freely. Ultrasonogram and CT-reconstructive urogram was done four weeks later, which revealed a 4 x 4 cm outpouching from the posterolateral wall of the urinary bladder with a narrow neck confirming the urinary bladder diverticulum. There was no evidence of stones, bladder wall thickening or tumour foci. Cystoscopy was done and findings were noted to be consistent with the CT urogram. Bladder neck incision was performed. As the patient was asymptomatic without any urinary symptoms, no further management was advised by the urologist. He is symptom free on a follow up of two years.

## Discussion

Bladder diverticulum is defined as herniation of the mucosa of the urinary bladder as a consequence of absence or weakness of the detrusor muscle. It is frequently seen in a trabeculated urinary bladder following bladder outlet obstruction. Rarely, due to increased intrabdominal pressure, the bladder diverticulum may herniate into the sac becoming its component. [1] Bladder diverticulum as a content in inguinal hernia surgery is a rare presentation (less than 0.1%). Levine first described inguinal hernia with bladder as content as scrotal cystocele in 1951 [2]. Most of the inguinal bladder hernias are diagnosed intraoperatively (77%), whereas 7% were diagnosed preoperatively and 16% postoperatively due to the complications. Bladder diverticulum can be congenital or acquired. Congenital component is rare and asymptomatic while the acquired component is always a pulsion diverticulum secondary to several etiological factors like bladder outlet obstruction. A patient with herniated bladder diverticulum can present with a wide variety of clinical presentations, ranging from an asymptomatic swelling in the inguinal region to lower urinary tract symptoms. The most distinctive finding in a bladder diverticulum is two-stage voiding. During micturition, the non-herniated part of the bladder empties quickly but the urine in the herniated part may require manual reduction [3]. However, in our case, as the patient was asymptomatic, the diagnosis was made intraoperatively. Previously cystography was considered as the gold standard in inguinal bladder hernias. However, multidetector CT is gaining importance as it gives better information on the surrounding structures and morphology of the anterior abdominal wall. An extraperitoneal or intraperitoneal approach can be employed for the surgical management of bladder diverticulum herniated through the inguinal canal. The surgical approach depends on the surgeon’s preference and the patient condition. In our case, we proceeded with the classical inguinal skin crease incision, which was extended once the diagnosis of bladder diverticulum as content was made. Manfredelli et al. reported a case of inguinal bladder diverticulum in a recurrent inguinal hernia in 2012, where surgical treatment was achieved by an infra-umbilical incision followed by reduction and fixation of the diverticulum [4]. Most case reports suggest diverticulectomy in this condition. However, as our patient was asymptomatic, this was deferred. In patients where the bladder diverticuli has developed as a result of benign prostatic hypertrophy, prostatectomy can be performed simultaneously. Bladder resection may be indicated in cases of bladder neck necrosis and bladder tumors. Nabavizadeh et al. reported a case of urinary bladder diverticulum herniation where cystoscopy was done prior to surgery to assess the location of the diverticular orifice [5]. One must be mindful of inadvertent bladder injury during surgery of hernias with this unusual content. Gurer et al. conducted a retrospective study on 1,950 patients who underwent groin hernia repair in their institution, of which 0.36% of the patients had urinary bladder as the content. Latrogenic bladder injury occurred in two patients [6].

## Conclusions

This case serves to report an acute presentation of urinary bladder diverticulum in obstructed inguinal hernia. The treatment of inguinal hernia remained the same irrespective of the content. The underlying cause has to be addressed in order to prevent recurrence. We recommend the combined management by a surgeon and a urologist to prevent inadvertent bladder injury in such rare cases. 

## References

[1] Fuerxer F, Brunner P, Cucchi JM, Mourou MY, Bruneton JN (2006). Inguinal herniation of a bladder diverticulum. Clin Imaging.

[2] Gomella LG, Spires SM, Burton JM, Ram MD, Flanigan RC (1985). The surgical implications of herniation of the urinary bladder. Arch Surg.

[3] Yong GL, Siaw MY, Yeoh AJ, Lee GE (2013). Inguinal bladder hernia: case report. Open J Urol.

[4] Manfredelli S, Zitelli A, Pontone S, Marcantonio M, Nargi A, Forte A, Angelici A (2012). An inguinal bladder diverticulum: case report of a rare finding in a recurrent inguinal hernia. Ann Ital Chir.

[5] Nabavizadeh R, Nabavizadeh B, Hampton LJ, Nabavizadeh A (2017). Herniation of a urinary bladder diverticulum: diagnosis and management of a fluctuating inguinal mass. BMJ Case Rep.

[6] Gurer A, Ozdogan M, Ozlem N, Yildirim A, Kulacoglu H, Aydin R (2006). Uncommon content in groin hernia sac. Hernia.

